# Vascular and Vasogenic Manifestations of Systemic ANCA-Associated Vasculitis with Renal Involvement in Non-Contrast Brain MRI in Patients with Acute Disease Onset

**DOI:** 10.3390/jcm11164863

**Published:** 2022-08-19

**Authors:** Arkadiusz Lubas, Jacek Staszewski, Artur Maliborski, Magdalena Mosakowska, Grzegorz Spłocharski, Anna Bilbin-Bukowska, Izabela Wołoszyńska, Renata Piusińska-Macoch, Daniel Pałka, Arkadiusz Zegadło, Stanisław Niemczyk

**Affiliations:** 1 Department of Internal Diseases Nephrology and Dialysis, Military Institute of Medicine, 04-141 Warsaw, Poland; 2 Department of Neurology, Military Institute of Medicine, 04-141 Warsaw, Poland; 3 Department of Radiology, Military Institute of Medicine, 04-141 Warsaw, Poland

**Keywords:** ANCA vasculitis, chronic kidney disease, central nervous system, cerebro-renal system

## Abstract

Background. Data concerning central nervous system (CNS) alterations in ANCA-associated vasculitis with renal involvement (AAVR) are sparse. The study aimed to assess vascular and vasogenic brain alterations in patients with acute onset of AAVR and the applicability of non-contrast magnetic resonance imaging (MRI) techniques in this diagnosis. Methods. Thirty-eight patients with acute onset of AAVR were included in the study. BVAS/WG, c-ANCA, p-ANCA, renal function and perfusion, neurological assessment, and brain MRI were performed. Results. Cerebral vascular alternating narrowing and dilatation (VAND) was detected in 42.1% of patients, and the black-blood was significantly more diagnostic than the TOF technique (*p* < 0.001). VAND occurrence was independently associated with the concentration of p-ANCA. The vasogenic white matter lesions (VWML) were found in 94.4% of patients, and in their detection, SWAN was significantly better than the FLAIR technique (*p* = 0.002). The number of VWML correlated with age and cranial nerve damage. Hemosiderin deposits were found in 21.6% of patients and were associated with a gait impairment and paresthesia. Conclusions. Vascular and vasogenic alterations in the CNS are frequent in patients with acute onset of systemic ANCA-associated vasculitis with renal involvement. Non-contrast MRI is useful in the diagnosis of brain vasculitis.

## 1. Introduction

Antineutrophilic cytoplasmic antibody (ANCA) associated vasculitis (AAV) is a systemic disease related to necrosing inflammation of small vessel walls. Although the definition of AAV comprises three diseases such as microscopic polyangiitis (MPA), granulomatosis with polyangiitis (GPA), and eosinophilic granulomatosis with polyangiitis (EGPA), their clinical signs often overlap [1]. However, recently published investigations suggest that the division of AAV due to the serotype of pathogenic antibodies could be more appropriate regarding epidemiology, clinical and laboratory features, and prognosis [2,3]. MPA (incidence 1–16/mln.) in 60% is associated with myeloperoxidase antibodies (p-ANCA), usually limited to the kidneys and expressed in 65% as rapidly progressive glomerulonephritis, seldom as the pulmonary-renal syndrome [4]. GPA (1.9–13/mln.) in about 75% is associated with proteinase-3 antibodies (c-ANCA) and expressed as granulomatous lesions in the upper pulmonary tract and lungs with up to 70% renal involvement. EGPA is most infrequent (1–3/mln.) and is driven by eosinophils and in 40% by ANCA (p-ANCA and/or c-ANCA) with the main involvement of lungs, upper airways, and peripheral nerves, whereas kidneys are affected in about 25%. The manifestations of acute kidney involvement in AAV include microscopic hematuria, proteinuria, and rapid kidney function decline [5]. The coexistence of ANCA justifies the diagnosis of ANCA-associated vasculitis with renal involvement (AAVR) and the start of immunosuppressive therapy induction even without a prior renal biopsy. In all forms of systemic AAV, secondary involvement of the central nervous system (CNS) is considered relatively rare. Although AAV can affect the nervous system in about 22–54% of patients, CNS involvement is estimated in less than 6.7–15% of cases [6]. Brain affection in AAV occurs in up to 10% of GPA, 1–8% of EGPA, and occasionally in MPA [7]. However, data concerning the occurrence of CNS involvement in AAV could be underestimated as an effect of diagnostic tests performed only due to the incidence of characteristic clinical symptoms. Vasculitis can affect both the CNS and/or the peripheral nervous system (PNS). The typical clinical PNS syndrome is mononeuropathy multiplex or asymmetric neuropathy, but distal-symmetric neuropathy can also be seen frequently. CNS vasculitis manifestation is usually not specific and includes, among others, focal or global symptoms such as paresis, aphasia, visual or gait disturbances, headache, cognitive dysfunctions, seizures, altered consciousness, and coma. The onset of symptoms can be acute, subacute, or chronic with a relapsing-remitting course. Both CNS and PNS symptoms in the course of vasculitis require careful and laborious clinical, neurophysiological, and electrophysiologic workouts and remain the most challenging disorders to diagnose and treat [8]. AAV can affect the brain in three ways: meningitis, pituitary gland granulomatous alterations, and vasculitis. While meningitis and pituitary gland alterations are more frequent in GPA, vasculitis can be recognized in all forms of AAV. Although secondary CNS involvement in the course of AAV probably does not worsen patients’ survival, it significantly influences and diminishes their quality of life [6]. In radiologic imaging methods, brain vascular lumen alterations manifest as longitudinal narrowing or alternating narrowing and dilatation so cold multiple stenoses and also as “the string of beads.” However, the concentric vascular wall thickening and enhancement in magnetic resonance imaging have recently been considered a hallmark of CNS vasculitis [9,10]. Vasogenic alterations encompass ischemic focal or diffuse white matter lesions (VWML)—hyperintense in magnetic resonance T2-weighted images in fluid-attenuated inversion recovery (FLAIR) and susceptibility-weighted angiography (SWAN) techniques, and hemorrhagic alterations (hemosiderin deposits of low signal intensity on SWAN images) most likely as a consequence of vascular wall inflammation [7]. The American College of Radiology Appropriateness Criteria recommends using head magnetic resonance angiography with or without intravenous contrast as the usually appropriate method for initial imaging in patients with suspected CNS vasculitis [11]. In this document, cervico-cerebral arteriography and head computed tomography angiography with intravenous contrast agent were estimated as having lower usability (“may be appropriate”) in diagnosing CNS vasculitis. Given the high burden of disease and difficulties in differential diagnosis, there is an urgent need for more precise diagnostic tools to enable earlier diagnosis and treatment. If the brain alterations during AAVR appear to be frequent, this could be the next issue helping the AAV activity and the treatment efficacy estimation.

The study aimed to assess the occurrence of vascular and vasogenic brain alterations in patients with acute onset of ANCA-associated vasculitis with renal involvement and the applicability of different techniques of non-contrast brain MRI in this diagnosis.

## 2. Materials and Methods

Study participants were recruited from August 2019 to November 2021, and all gave their signed informed consent. Patients with a flare of ANCA-associated vasculitis with renal involvement qualified for the induction therapy were included in the study. Inclusion criteria were age > 18 years and signed agreement for the study. The exclusion criteria comprised disorders of consciousness, contraindications for brain MRI, cardiac or pulmonary insufficiency, symptoms of an active viral or bacterial infection, active oncological state, and advanced performance status impairment (ECOG/WHO performance status ≥ 3 [12]).

### 2.1. Clinical Assessment

A profound medical documentation analysis with an anamnesis, including duration and clinical signs of AAV, was performed. Results were searched, and patients were asked for actual symptoms of ear, nose, and throat (ENT) involvement, ocular aberrations, and skin alterations. Pulmonary signs considered hemoptysis, lung nodular enlargement, and alveolar hemorrhages. Gastrointestinal involvement was considered if abdominal pain, nausea, vomiting, diarrhea, hemorrhage, ulcers, peritonitis, or bowel perforation occurred. Moreover, joint complaints were recorded. Lastly, AAV activity in Birmingham Vasculitis Activity Score for Wegener’s granulomatosis (BVAS/WG) scale was assessed [13].

### 2.2. Neurological Assessment

A structured medical history regarding past neurological examinations (e.g., available tests of electroneurography (ENG) and electromyography (EMG)), diagnoses, and complaints, including a history of strokes and transient ischemic attacks (TIA), mono- or polyneuropathy, myopathy, chronic headaches, gait imbalance, encephalopathy, seizures, loss of sensation, muscle weakness was taken. The physical examination of the neurological system comprised of an assessment of both the CNS and PNS performed by the same two board-certified senior neurologists. It covered mental status, cranial nerves, motor system, reflexes, sensory system, coordination, and gait assessments. Based on the neurological, ENG, and EMG examinations, PNS dysfunction was recognized in the case of peripheral mononeuropathy, multiple mononeuropathies, polyneuropathy, or cranial neuropathy symptoms, while CNS dysfunction was recognized in the case of corticospinal, cerebellar, or brainstem signs. ENG and EMG tests were performed according to the standard protocol [14].

### 2.3. Neuropsychological Assessment

A trained neuropsychologist performed assessments on cognitive functions and depression. The tests included measures of global cognitive function (the Mini-Mental State Examination (MMSE)—Standardized) and the Hamilton Depression Rating Scale (HDRS) [15,16]. Scores ≤ 7 in HDRS represented the absence of depression, while a score ≤ 26 in MMSE adjusted for age and education was used as a cut-off for cognitive decline [17].

### 2.4. Laboratory Tests

Blood tests assessing hemoglobin (Hgb [g/dL]), kidney function (creatinine [mg/dL], urea [mg/dL]) active inflammation (high-sensitive C-reactive protein (hsCRP) [mg/dL], procalcitonin (PCT) [ng/mL]) and antineutrophil antibodies p-ANCA and c-ANCA [IU/mL] were performed. In addition, urinary albumin excretion ratio (UACR) [mg/g] was estimated from the second morning spot urine.

### 2.5. Renal Perfusion

We used the Logiq P6 (GE Healthcare, Seoul, Korea) ultrasound equipment with a 4 L (2–5 MHz) convex transducer to scan and record 3–5 s of color Doppler clips showing renal cortical blood flow, as it was described earlier [18]. Clips were assessed in the medical device (PixelFlux, Chameleon Software, Leipzig, Germany). Then the average intensity of arterial cortical blood flow as the renal cortical perfusion (RCP) [cm/s] and renal cortical arterial area (RCAA) [cm^2^] were estimated [19,20].

### 2.6. Brain Magnetic Resonance Imaging

Brain MRI (3-Tesla unit; Discovery MR750W (GE Healthcare, Florence, SC, USA) with a 12-channel head coil) was performed just before the induction of immunosuppressive therapy. To avoid occasional contrast media infusion regarding kidney insufficiency, we decided to use non-contrast MRI with several widely accessible techniques: black-blood (BB) (pulse gated (PG), short tau inversion recovery (STIR), fast spin echo (FSE), axial), time of flight (TOF), diffusion-weighted imaging (DWI), T2 FLAIR FSE axial, SWAN, 3D Cube T2 Iso FSE sagittal, and 3D Cube T1 Iso FSE sagittal, that were recommended to the brain and vascular assessment [21,22,23]. The imaging parameters for used sequences are presented in Table 1. Two board-certified senior radiologists reviewed the achieved images. The BB technique shows the blood within vessels in black, enabling identification of the vessel lumen. 3D TOF angiography technique is helpful in the luminal vessel contour assessment and performed in 1.5 Tesla MRA is comparable to digital subtraction angiography [9,24]. MRI signs of vasculitis such as focal and longitudinal narrowing, vascular alternating narrowing and dilatation (VAND), and stenoses were identified and counted. The focal, hyperintense in T2-weighted sequences, white matter lesions (VWML), and low signal intensity on SWAN images hemosiderin deposits were recognized as vasogenic alterations.

### 2.7. Statistical Analysis

The results of investigated parameters were presented in mean with standard deviation and median with interquartile range (IQR). In the case of categorical variables, results were presented as a number with occurrence frequency. Pearson or Spearman tests were used according to the distribution normality of investigated variables for correlation analysis. Otherwise, point-biserial analysis for nominal and continuous variables was performed. Differences between categorical variables were checked with the chi-squared test and between continuous dependent variables with the Wilcoxon’s test. The pairwise deletion was used if missing data occurred. To investigate the independent associations, the multivariable backward logistic regression analysis was performed. The two-tailed *p*-value < 0.05 was considered statistically significant. For statistical analysis, Statistica 12 (StatSoft Inc., Cracow, Poland) software was used.

## 3. Results

Thirty-eight patients (21F, 17M, age 61.4 ± 10.8) with acute onset of ANCA-associated vasculitis (31 with predominant p-ANCA and 7 with c-ANCA) with renal involvement qualified for the induction immunosuppressive therapy were included in the study. Eleven patients were treated with hemodialysis at the time of the inclusion. Four patients had a history of vasculitis in the past, but only two of them had been treated with prednisone (5 mg daily) and one with mycophenolate mofetil (250 mg daily) as a maintenance regimen. No more patients had been treated immunosuppressively. Four patients were treated with anticoagulants, and five patients were treated with antiplatelet drugs. The demography, disease activity, and blood test results are presented in Table 2. Results of the clinical and neurological assessments are shown in Table 3. The mean MMSE score was 25.1 ± 2.1, and the HDRS was 6.1 ± 1.4.

### 3.1. Vascular Alterations

**Figure 1:**
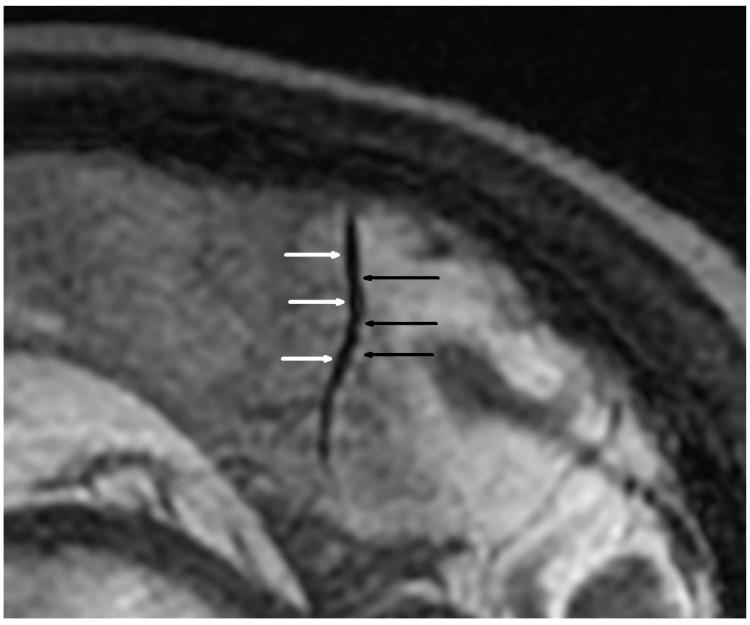
Alternating narrowing and dilatation in the intracranial vessel (sagittal plane). 3D Cube T2 Iso FSE sagittal sequence; black arrow—narrowing; white arrow—widening.

**Figure 2:**
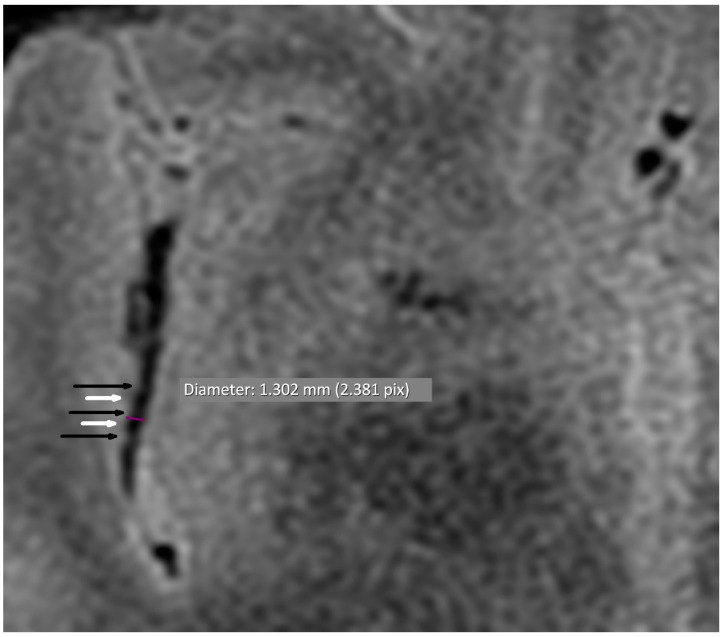
Alternating narrowing and dilatation in the intracranial vessel (axial plane). BB STIR T2 axial sequence; purple—diameter; black arrow—narrowing; white arrow—widening.

Cerebral MRI in TOF scanning revealed five microaneurysms (dimensions from 1.7 × 1.8 mm to 3.5 × 4.0 mm), and two of them were localized in the right internal carotid arteria. We found only two segmental arterial narrowing in the investigated group. The occurrence of VAND was detected in 16/38 (42.1%) patients (Table 4, Figure 1 and Figure 2). Considering the different quantities of VAND, the BB MRI technique was more diagnostic than the TOF sequence (*p* < 0.001).

The occurrence of VAND in secondary branches correlated significantly with age and concentration of p-ANCA but not with BVAS score and other parameters (Table 5). However, VAND findings in the lower diameter tertiary branches correlated significantly with pulmonary alterations and the p-ANCA concentrations.

In multivariable backward logistic regression analysis, including age, BVAS score, p-ANCA concentration, pulmonary involvement, and ataxia, only age (OR 0.891, 95% CI: 0.812–0.977; *p* = 0.014) and p-ANCA (OR 1.023, 95% CI: 1.005–1.040; *p* = 0.010) were independently associated with VAND in secondary arterial branches. Moreover, considering the same model, the logistic regression analysis performed for VAND in tertiary arterial branches showed that only the p-ANCA concentration is associated with these alterations (OR 1.017, 95% CI: 1.003–1.031; *p* = 0.015).

The renal function and perfusion parameters did not correlate with cerebral vessels alterations. However, RCAA was significantly associated with BVAS (r = −0.34; *p* = 0.042) and almost with c-ANCA (r = −0.34; *p* = 0.056), and conversely RCP correlated with c-ANCA (r = −0.40; *p* = 0.021) and nearly with BVAS (r = −0.30; *p* = 0.077). Moreover, creatinine was associated with p-ANCA (r = 0.50; *p* = 0.001).

### 3.2. Vasogenic Lesions

The vasogenic lesions in the cerebral white matter (VWML), hemosiderin increments, and diffusion restriction were estimated. VWML were found in 94.4% of patients. The exact results of cerebral MRI are presented in Table 6.

The number of VWML detected in FLAIR correlated significantly only with creatinine (r = −0.33; *p* = 0.042) and almost significantly with age (r = 0.31; *p* = 0.062). In eight participants, 37 deposits of hemosiderin (max. length of 4.5 mm) were indicated. Significant associations of VWML detected in SWAN and hemosiderin deposits are presented in Table 7. The anticoagulant or platelet therapy was not associated with VWML or hemosiderin deposits number and occurrence.

MRI scans also revealed three focal restrictions of the diffusion (max. dim.: 4 × 7 mm), located in subcortical white matter (1 focus) and semioval center (2 foci) in two patients.

## 4. Discussion

Our findings show new evidence of CNS involvement in systemic AAV. Moreover, we showed the noninvasive brain imaging method’s applicability in diagnosing alterations of small cerebral vessels. Using 3T-MRI irrespective of clinical symptoms, we found that angiographic features of CNS vasculitis are much more frequent (42%) in patients with a flare of AAVR than was reported in those with clinical suspicion of CNS involvement. Our data confirm this statement because, in a neurologist’s assessment, symptoms of CNS dysfunction were estimated only at 26%. Performed 3T-MRI with the BB instead of TOF MRI technique confirmed CNS vasculitis in 42% of patients. Our findings appear to be of great importance because ENT involvement was up to 40% in the investigated patients, which means that CNS AAV involvement is even slightly higher. This suggests that a routine MRI diagnosis of brain involvement during AAVR can significantly benefit the disease activity estimation. Moreover, this diagnostic procedure has the potential for treatment efficacy monitoring, but this should be investigated in future studies.

Harsha et al. investigated histologically proven small vessel brain vasculitis and found 43 hyperintense lesions in T2/FLAIR and SWAN techniques in three patients with lymphocytic vasculitis [25]. However, they used contrast intravenous agents, contraindicated in dialyzed patients. Performing high-resolution 3T MRI with gadolinium contrast media in 13 patients with primary CNS vasculitis, Obusez et al. found arterial wall thickening and wall enhancement in 12 participants [21]. All 9 patients (100%) with concentric wall thickening, which is thought to be a hallmark of CNS vasculitis, had vascular lumen alterations in the form of focal stenosis, irregular narrowing, and occlusion. However, of the three patients with eccentric wall thickening, only one had narrowing and stenosis. These data suggest that 10/13 (77%) patients presented with primary CNS vasculitis, from which 10/12 (83%) with vascular wall thickening had vascular lumen alterations (main vascular pathology in our study). Moreover, in contrast to Obusez et al. study, which could assess alterations in primary arterial branches, we used 3T MRI BB technique but without gadolinium contrast media to avoid nephrogenic systemic fibrosis, especially in dialyzed patients, and found arterial lumen alterations in secondary and tertiary vascular branches. This suggests comparable or even better usability of non-contrast 3T MRI in diagnosing brain vasculitis. This hypothesis could be supported by Sundaram et al. study, which used high-resolution 3T magnetic resonance vascular wall imaging, and reported the lack of arterial wall thickening in 7/20 (35%) patients with primary CNS vasculitis [10].

In our study, PNS dysfunction was found in almost 46% of patients, and PNS was the third most frequently affected organ (after kidneys—100% and lungs—63%). Our results demonstrated the reversed association between PNS dysfunction or ataxia and VWML and VAND in secondary arterial branches, respectively. These could be related to the predominant involvement of the peripheral circulation (e.g., nerve vessels), leading to thrombosis and subsequently to peripheral neuropathy with proprioceptive sensory pathway dysfunctions in that group of patients. We also did not find any significant associations between CNS symptoms and MRI markers, which was somewhat unexpected because almost all participants (94%) had VWML, which can result from hypoperfusion and ischemia. This lack of correlation could be related to the disease’s slow progression in most patients, which may favor “silent” neurological manifestations or be related to different endogenous mechanisms of CNS damage. These could include both latent cerebral infarctions due to cerebral vasculitis and/or direct neurotoxic effects of inflammatory cytokines, but the actual mechanism remains uncertain [26].

We showed that age and p-ANCA are independently related to VAND in secondary vascular branches. Although the presence of p-ANCA can induce vasculitis and is a known marker of AAV activity, an association with brain vascular alterations typical for this disease is rather expected. However, the correlation between brain vasculitis and age can result from prolonged exposure to risk factors such as smoking, silica dust, and infections [4]. This could be the reason that GPA and MPA occur mainly in older people.

Although we did not find any significant association between VAND, VWML, hemosiderin deposits, and cognitive impairment, almost 77% of the patients fulfilled the criteria for this diagnosis. This observation can lead to the hypothesis that vascular and vasogenic brain injury during AAV can be associated with the development of cognitive impairment. However, a study on a more extensive group is required to confirm this connection.

Considering the kidney involvement during AAV in all our study patients, the common occurrence of brain VWML (94% of patients), and the fact that 42% of the patients were diagnosed with VAND, we could expect a correlation in the cerebro-renal axis. Nevertheless, kidney function and renal cortical perfusion were not directly associated with signs of brain involvement. This discrepancy can be related to the different time points and the magnitude of the involvement of particular organs. However, we found significant correlations of renal cortical perfusion parameters with BVAS and c-ANCA, and between VAND and BVAS and p-ANCA, which expresses AAV activity. Moreover, renal function was associated with p-ANCA. By the similarity of structure and contents of small vessels and amount of organ blood flow, the same endothelium kidneys and brain can share the same vascular damage risk factors (cerebro-renal system), and vascular disease, especially vasculitis, can affect both [27,28]. Thus, the presumption that renal function and perfusion are connected with vascular and vasogenic brain alterations by the extended time of the AAV activity seems justified.

Despite promising results, our study has several limitations. First, we included patients with recognized flare/acute onset of AAV with renal involvement who were qualified for immunosuppressive induction therapy with no respect to the duration of the disease. Moreover, due to the spread in time complex diagnostic procedures, some investigations could be omitted in particular patients. For example, only 21 patients in the study group could have had ENG and EMG examinations, and only 14 patients underwent neuropsychological assessments. The abovementioned data loss was mainly related to COVID-related organization troubles. These drawbacks could have influenced the results of the frequency of cognitive impairment, depression, or subclinical neuropathy. However, considering the careful physical neurological examination, we believe that the PNS dysfunction rate is accurate. In addition, the neurological and neuropsychological spectrum of the disease was also not the primary aim of the present work, but it will be focused on in future studies.

## 5. Conclusions

Vascular and vasogenic alterations in the central nervous system are frequent in patients with an acute onset of systemic ANCA-associated vasculitis with renal involvement. Non-contrast MRI techniques, especially black-blood and SWAN sequences, are helpful in the detection of cerebral vessels and vasogenic pathology in these patients. The routine non-contrast brain MRI would benefit the AAV activity assessment. Although renal function and perfusion can be associated with the vasculitis-related brain alterations in the course of AAV by the activity of the disease, the occurrence of specific organ involvement symptoms at a different time made it challenging to prove.

## Figures and Tables

**Table 1:** MRI sequence parameters.

	Freq FOV (cm)	Phase FOV (mm)	Slice Thickness (mm)	Spacing (mm)	TR (ms)	TE (ms)	TI (ms)	Freq Matrix	Phase Matrix
T2 FLAIR FSE Axial	24	0.8	3	0.5	9000–11,000	120–135	2461–2645	320	200
3D Cube T2 Iso FSE Sagital	24	1	0.8	0	3000	94–104	-	288	288
3D Cube T1 Iso FSE Sagital	24	1	0.8	0	500	18–20	-	320	320
SWAN	24	1	0.8	0	43–57	22–23	-	288	288
PG Dark Blood STIR FSE Axial	14	1	2	0	1520–2400	41–44	180	224	192
3D TOF FS Axial	20	0.75	0.5	0	23–24	3.4–6.9	-	400	400
DWI B1000	24	1	3	0.5	11,500–15,500	107–110	-	160	160

DWI—diffusion-weighted imaging; FLAIR—fluid-attenuated inversion recovery; FS—fat saturation; FSE—fast spin echo; MRI—magnetic resonance imaging; PG—pulse gated; STIR—short tau inversion recovery; SWAN—susceptibility-weighted angiography; TOF—time of flight; FOV—field of view; TR—repetition time; TE—echo time; TI—inversion time.

**Table 2:** Demographical and clinical data with blood and serum test results in the investigation group.

	Mean	STD	Median	IQR
Age [y]	61.4	10.8	62.1	17.6
Disease duration [mths]	12.3	23.0	2.9	10.5
BVAS	7.53	3.21	7	4
Hgb [g/dL]	9.98	1.89	9.5	2.3
Creatinine [mg/dL]	3.97	2.64	3.35	3.3
Urea [mg/dL]	116.21	69.16	117.5	75
UACR [mg/g]	1.91	1.76	1.23	2.43
RCP [cm/s]	0.222	0.230	0.146	0.131
RCAA [cm^2^]	0.193	0.146	0.171	0.118
hsCRP [mg/dL]	1.81	2.64	0.845	1.21
PCT [ng/mL]	0.25	0.30	0.175	0.195
p-ANCA [IU/mL]	50.94	56.57	24	107.9
c-ANCA	15.75	43.44	0.2	0.5

BVAS—Birmingham Vasculitis Activity Score; c-ANCA—proteinase 3—anti-neutrophil cytoplasmic antibody; p-ANCA—myeloperoxidase-anti-neutrophil cytoplasmic antibody; Hgb—hemoglobin; hsCRP—high-sensitive C-reactive protein; IQR—interquartile range; PCT—procalcitonin; RCAA—renal cortical artery area; RCP—renal cortical perfusion; STD – standard deviation; UACR—urinary albumin to creatinine ratio.

**Table 3:** Results of the clinical and neurological assessment.

	Number/Patients	Frequency (%)
**Clinical assessment**		
ENT (ear, nose, throat)	15/38	39.5
Ocular	7/38	18.4
Cutaneous	11/38	28.9
Pulmonary	24/38	63.2
-alveolar hemorrhage	7/38	18.4
-nodules	21/38	55.3
-hemoptysis	8/38	21.1
Gastrointestinal	5/38	13.2
Joint	10/38	26.3
Cardiovascular	1/38	2.6
**Neurological assessment**		
Chronic headaches *	8/37	21.6
Gait imbalance *	9/37	24.3
Seizures *	0/37	0.0
Encephalopathy *	0/37	0.0
Legs and/or arms paresthesia *	10/37	27.0
Sensory and/or motor polyneuropathy *	10/37	27.0
Stroke or TIA *	0/37	0.0
Myopathy *	0/37	0.0
Cranial neuropathy	2/37	5.4
Mononeuritis/Mononeuritis multiplex	9/21	42.9
Polyneuropathy	10/21	47.6
Ataxia	16/37	43.2
Pyramidal symptoms	8/37	21.6
Extra-pyramidal symptoms **	9/37	24.3
CNS dysfunction	10/37	26.3
PNS dysfunction	17/37	45.9
Cognitive impairment	10/13	76.9
Depression	3/14	21.4

CNS—central nervous system; PNS—peripheral nervous system; TIA—transient ischemic attack; * in anamnesis, ** hands tremor (9/9).

**Table 4:** Results of the cerebral MRI angiography in the investigation group.

	Black-Blood	Time of Flight	*p*-Value
	Number (%)	Number (%)	
Segmental narrowing in secondary vascular branches	1/38 (2.6)	2/38 (5.3)	0.556
Segmental narrowing in tertiary vascular branches	0/38 (0.0)	0/38 (0.0)	1.0
Alternating narrowing and dilatation in secondary vascular branches	13/38 (34.2)	1/38 (2.6)	<0.001
Alternating narrowing and dilatation in tertiary vascular branches	13/38 (34.2)	1/38 (2.6)	<0.001
Alternating narrowing and dilatation in secondary and tertiary vascular branches	16/38 (42.1)	2/38 (5.3)	<0.001

**Table 5:** Correlations between alternating narrowing and dilatation in secondary and tertiary vascular branches and clinical and blood test results.

	Alternating Narrowing and Dilatation in the Secondary Vascular Branches	Alternating Narrowing and Dilatation in the Tertiary Vascular Branches
Age [y]	−0.349 *	−0.162
Gender [M]	0.021	0.132
Disease duration [months]	−0.204	−0.187
BVAS [points]	0.178	0.283 ^#^
ENT (ear, nose, throat) [n]	−0.015	−0.128
Ocular [n]	−0.056	0.230
Cutaneous [n]	0.029	0.029
Pulmonary [n]	0.206	0.321 *
Gastrointestinal [n]	0.048	0.048
Joint [n]	0.199	0.073
Cardiovascular [n]	−0.119	−0.119
Hgb [g/dL]	−0.109	0.084
Creatinine [mg/dL]	0.038	−0.025
Urea [mg/dL]	0.022	0.049
UACR [mg/g]	0.139	0.163
RCP [cm/s]	−0.027	−0.059
RCAA [cm^2^]	−0.133	−0.117
hsCRP [mg/dL]	−0.113	0.004
PCT [ng/mL]	−0.010	−0.044
p-ANCA	0.323 *	0.379 *
c-ANCA	−0.198	−0.198
CNS dysfunction [n]	0.062	0.062
PNS dysfunction [n]	−0.224	−0.244
Gait imbalance [n]	0.111	0.111
Ataxia [n]	−0.300 ^#^	−0.300 ^#^
Cognitive impairment [n]	−0.318	−0.318
Depression [n]	0.318	−0.058
Antiplatelet therapy [n]	0.047	0.047
Anticoagulation therapy [n]	−0.067	−0.067

BVAS—Birmingham Vasculitis Activity Score; c-ANCA—proteinase 3—anti-neutrophil cytoplasmic antibody; pANCA—myeloperoxidase-anti-neutrophil cytoplasmic antibody; Hgb—hemoglobin; hsCRP—highly sensitive C-reactive protein; CNS—central nervous system; PNS—peripheral nervous system; PCT—procalcitonin; RCAA—renal cortical artery area; RCP—renal cortical perfusion; UACR—urinary albumin to creatinine ratio. *—significant correlation (*p* < 0.05); ^#^—0.05 ≤ *p* < 0.10.

**Table 6:** Comparison of the occurrence and quantity of vasogenic white matter lesions in FLAIR and SWAN MRI techniques in patients with ANCA-associated vasculitis with renal involvement.

	FLAIR		SWAN		*p*-Value
	Number (%)	Median (IQR)	Number (%)	Median (IQR)	
Presence of white matter vasogenic lesions	34/38 (89.5)		34/36 (94.4)		0.434
Number of white matter vasogenic lesions		21.0 (36.0)		23.0 (38.0)	0.002

ANCA—anti-neutrophil cytoplasmic antibody; FLAIR—fluid-attenuated inversion recovery; IQR—interquartile range; MRI—magnetic resonance imaging; SWAN—susceptibility-weighted angiography.

**Table 7:** Significant correlations of vasogenic alterations.

	VWML (SWAN)		Hemosiderin Deposits	
	Occurrence	Number	Occurrence	Number
Age [y]		0.348		
Gender [M]			0.314 ^#^	
Urea [mg/dL]			0.293 ^#^	
p-ANCA [IU/mL]			0.297 ^#^	0.271 ^#^
Alveolar hemorrhage [n]	−0.494			
Gastrointestinal involvement [n]				0.287 ^#^
Gait impairment [n]			0.530	0.578
Legs and/or arms paresthesia [n]			0.483	0.338
PNS dysfunction [n]	−0.284 ^#^			
Cranial neuropathy [n]		0.609		

PNS—peripheral nervous system; *p*-ANCA—myeloperoxidase-anti-neutrophil cytoplasmic antibody; SWAN—susceptibility-weighted angiography; VWML—vasogenic white matter lesions; ^#^—0.05 ≤ *p* < 0.10.

## Data Availability

The dataset is with the authors and available on request.
